# Oxidative Stress Enhances the TGF-β2-RhoA-MRTF-A/B Axis in Cells Entering Endothelial-Mesenchymal Transition

**DOI:** 10.3390/ijms23042062

**Published:** 2022-02-13

**Authors:** Katarzyna Sobierajska, Marta E. Wawro, Jolanta Niewiarowska

**Affiliations:** Department of Molecular Cell Mechanisms, Medical University of Lodz, 92-215 Lodz, Poland; marta.wawro@umed.lodz.pl

**Keywords:** MRTFs, EndMT, oxidative stress, fibrosis, TGF-β

## Abstract

Around 45% of deaths in the EU and the US are due to fibrotic diseases. Although myofibroblasts are detected in various fibrotic tissues, they are mostly transdifferentiated from endothelial cells during the endothelial-mesenchymal transition (EndMT) induced by tumor growth factor-beta (TGF-β) family members. Growing evidence indicates that oxidative stress might enhance the sensitivity and the effects of TGF-β stimulation; however, the molecular mechanisms involved in the coordination of oxidative stress and TGF-β inductions remain poorly understood. Our findings indicate for the first time that oxidative stress enhances mesenchymal trans-differentiation of human microvascular endothelial cells (HMEC-1 cells) and that the oxidative stress-dependent TGF-β2-RhoA/Rac1-MRTF-A axis is critical for the induction of later stages of EndMT. This additive effect was manifested in TGF-β1-stimulated and Snail-overexpressed cells, where it caused higher cell elongation and faster migration on collagen I layers. Additionally, Western blot assay indicated the presence of alterations in cell contraction and EndMT markers. We conclude that complex anti-fibrotic therapies based on the inhibition of MRTF activities and oxidative stress might be an attractive target for fibrosis treatment.

## 1. Introduction

Fibrosis is characterized by the excessive accumulation of extracellular matrix proteins secreted by myofibroblasts in response to cellular damage. It commonly occurs due to chronic inflammation, which can finally lead to organ dysfunction [1]. While fibrotic disease progression is estimated to be responsible for 45% of deaths in the United States of America and the European Union [2], little is known of the molecular mechanisms responsible for its development.

Since myofibroblasts play a primary role in the pathogenesis of fibrosis, a growing number of studies are focused on understanding elements of their biology, such as their origin and phenotypic differences. One of the crucial precursors of myofibroblast is endothelial cells (ECs), which undergo an endothelial-mesenchymal transition (EndMT) under the influence of cytokines and growth factors [3], mainly tumor growth factor-beta (TGF-β) family members [4,5]. EndMT-induced ECs demonstrate downregulation of endothelial markers like VE-cadherin, claudin, and zona-occludens 1 (ZO-1) and increased expression of typical mesenchymal markers, such as Fibroblast-specific protein 1 (FSP-1), alpha-smooth muscle actin (α-SMA), and N-cadherin. This process is regulated by transcription factors such as Snail, Slug, and Zinc Finger E-Box Binding Homeobox 1 (ZEB-1), whose levels are elevated during cellular transdifferentiation [6]. Furthermore, cell–cell connections are disrupted, and the promigratory abilities of the cells are increased. Despite numerous studies, the mechanism of action of particular TGF-β forms remains unclear and seems to be dependent on cell origin [4,5]. It has been demonstrated that, in contrast to TGF-β1, with affected only the early stages of EndMT, TGF-β2 stimulates HMEC-1 cells to take a more elongated shape and significantly increase their modulation of endothelial and mesenchymal marker levels [5].

The role of the TGF-β family members in fibrosis development has been intensively examined in the past years. Studies suggest that oxidative stress promotes TGF-β-induced fibrosis [7]. Some TGF-β-dependent pathways stimulate the production of reactive oxygen species (ROS), which activates and mediates many of the TGF-β-dependent fibrogenic effects observed in non-phagocytic cells, such as epithelial, endothelial and smooth muscle cells, and fibroblasts [8,9]. Despite growing evidence indicating that TGF-β and ROS cooperate in fibrogenesis development, the molecular mechanisms involved in these processes are primarily undetermined.

The principal mechanism regulating cellular transdifferentiation is cytoskeleton remodeling. Changes in cell shape and elongation and the modulation of adhesion and migration properties observed during EndMT are controlled by the small G-proteins belonging to the Rho GTPase family. This group consists of about 20 proteins, four of which (RhoA, RhoB, Rac1, and cdc42) are activated by TGF-β-dependent pathways [10,11]. The activation of the Rho family and the remodeling of cell structures affect the formation of stress fibers along the long axis of the myofibroblast, which increases the contraction ability characteristic for fibrosis tissue [12]. The G-actin-myocardin-related transcription factor A or B (MRTF-A/B)–serum response factor (SRF) pathway regulates the expression of most of the proteins associated with the cytoskeleton and its reorganization. The G-actin/MRTF-A/B complex formed in the cytoplasm under physiological conditions disassociates during cell stimulation. MRTF-A and MRTF-B translocate to the nucleus and bind to the SRF as the co-activator, altering the expression of numerous cytoskeletal-associated proteins (FAK, vinculin, and α-SMA) [13]. Additionally, during mesenchymal transdifferentiation, MRTFs regulate the expression of zinc-finger proteins, such as Snail and Slug [14].

The present study evaluates the role of TGF-β family members in the mesenchymal transdifferentiation of human microvascular endothelial cells (HMEC-1 cells) under oxidative stress conditions. Our findings indicate that the MRTF-regulated development of fibrosis and that cooperation between MRTF-A and MRTF-B play critical roles in the induction of the advanced stages of EndMT. They also demonstrate that control of MRTF represents a potential target for anti-fibrotic therapies.

## 2. Results

### 2.1. Hydrogen Peroxide Enhances the TGF-β Effect of EndMT Stimulation HMEC-1 Cells

**Figure 1:**
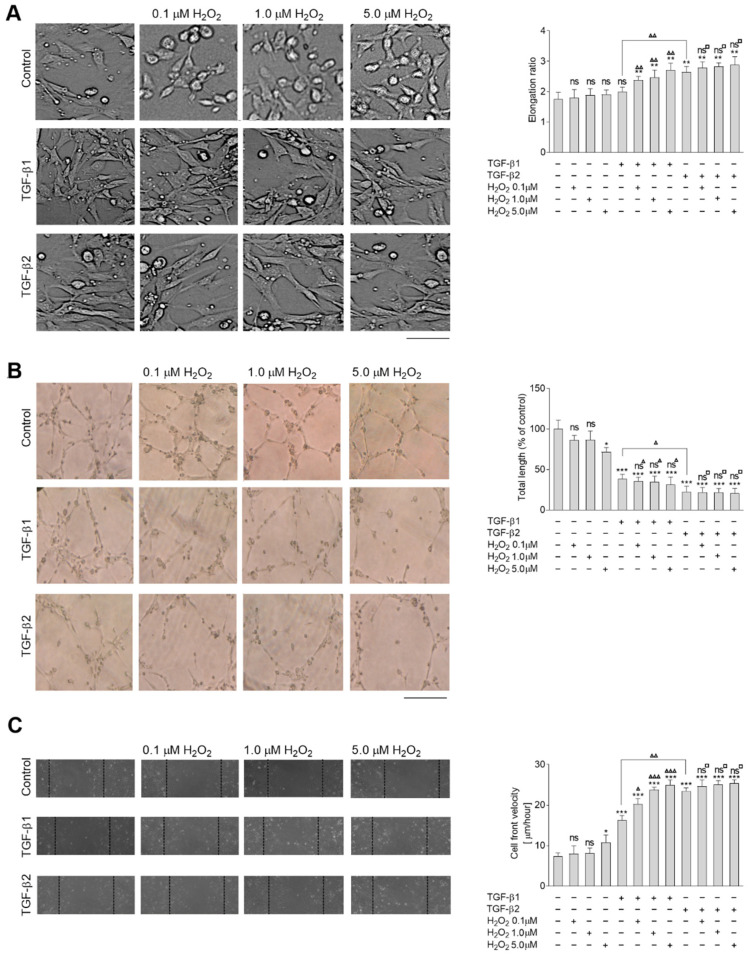
Co-stimulation by TGF-β1 or TGF-β2 and H_2_O_2_ induce later stages of EndMT in HMEC-1 cells. HMEC-1 cells were treated with TGF-β1 or TGF-β2 (10 ng/mL) and 0, 0.1, 1.0, or 5.0 µM H_2_O_2_ or only with H_2_O_2_ for 48 h. (**A**) Morphology was analyzed by DIC microscopy. At least 50 cells were measured to calculate the elongation ratio. Representative images are shown, with the graph displaying means (*n* = 3) ± SD; ** *p* < 0.01 in comparison to control cells, ▲▲ *p* < 0.01 in comparison to TGF-β1-treated cells, ns—not significant in comparison to control cells; ns^□^—not significant in comparison to TGF-β2-treated cells. Scale bar 100 µm. (**B**) Capillary formation was analyzed in Matrigel™. An appropriate number of cells were placed on Matrigel™, and after six hours, the tube formation was observed under a microscope at ×40 magnification and photographed. Representative images are shown and quantitative analysis of mean tube lengths from three independent experiments were made in ImageJ (*n* = 3) ± SD; * *p* < 0.05, *** *p* < 0.001 in comparison to control cells, ns—not significant in comparison to control cells; ▲ *p* < 0.05 in comparison to TGF-β1-treated cells ns^▲^—not significant in comparison to TGF-β1-treated cells, ns^□^—not significant in comparison to TGF-β2-treated cells. Scale bar 100 µm. (**C**) The wound healing properties were analyzed. An appropriate number of cells was seeded on 12-well plates pre-coated with collagen I. After two days, a monolayer of confluent cell layer was wounded and images were captured 8 h later. Results are given as a standard error of the mean of cell front velocity (*n* = 3). Statistical significance in all experiments was marked: * *p* < 0.05, *** *p* < 0.001 in comparison to control cells, ▲ *p* < 0.05 in comparison to TGF-β1-treated cells, ▲▲ *p* < 0.01 in comparison to TGF-β1-treated cells, ▲▲▲ *p* < 0.001 in comparison to TGF-β1-treated cells, ns—not significant in comparison to control cells, ns^□^—not significant in comparison to TGF-β2-treated cells.

To examine the role of oxidative stress with EndMT stimulation, HMEC-1 cells were incubated with 10 ng/mL TGF-β1 or TGF-β2 under 0.1, 1.0, or 5.0 µM H_2_O_2_ pressure. It has been found that TGF-β2 induces EndMT more strongly than TGF-β1, with their resulting respective elongation ratios being approximately 50% and 15% higher [15]. Co-stimulation with H_2_O_2_ resulted in the cells becoming longer in a concentration-dependent manner, with estimated elongation ratios ranging from 19% (0.1 µM H_2_O_2_) to 36% (5 µM H_2_O_2_) in TGF-β1-stimulated cells (Figure 1A). In contrast, H_2_O_2_ induction resulted in only slightly observed changes in TGF-β2-treated cells. The elongation ratio increased from 5% (0.1 µM H_2_O_2_) to less than 10% (5 µM H_2_O_2_), whereas the effect of H_2_O_2_ stimulation alone resulted in between 4% (0.1 µM H_2_O_2_) to nearly 8.5% cell elongation (5 µM H_2_O_2_). The mesenchymal-like cells demonstrated a more elongated cell shape (following TGF-β1 or TGF-β2 treatment), which correlated with a decrease in capillary formation (Figure 1B). Maximal H_2_O_2_ concentration (5.0 µM H_2_O_2_) resulted in a 30% decrease in capillary length. Marked alterations of capillary formation were seen after TGF-β1 or TGF-β2 stimulation (60% or 75% decreases of capillary length, respectively). Although H_2_O_2_ had a concentration-dependent effect on cells treated with TGF-β1, it was not detected following TGF-β2 treatment. Cells treated with TGF-β1 demonstrated twice the rate of cell migration on collagen type I compared to TGF-β2, and co-treatment with H_2_O_2_ (5 µM) increased migration speed by two and half times compared to the untreated cells, i.e., similar to the speed of the TGF-β2-stimulated cells. Co-treatment with H_2_O_2_ did not affect the TGF-β2-treated cells (Figure 1C). 

**Figure 2:**
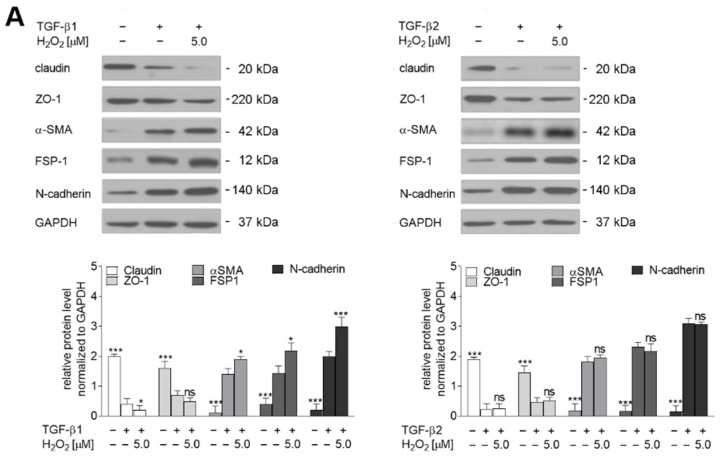

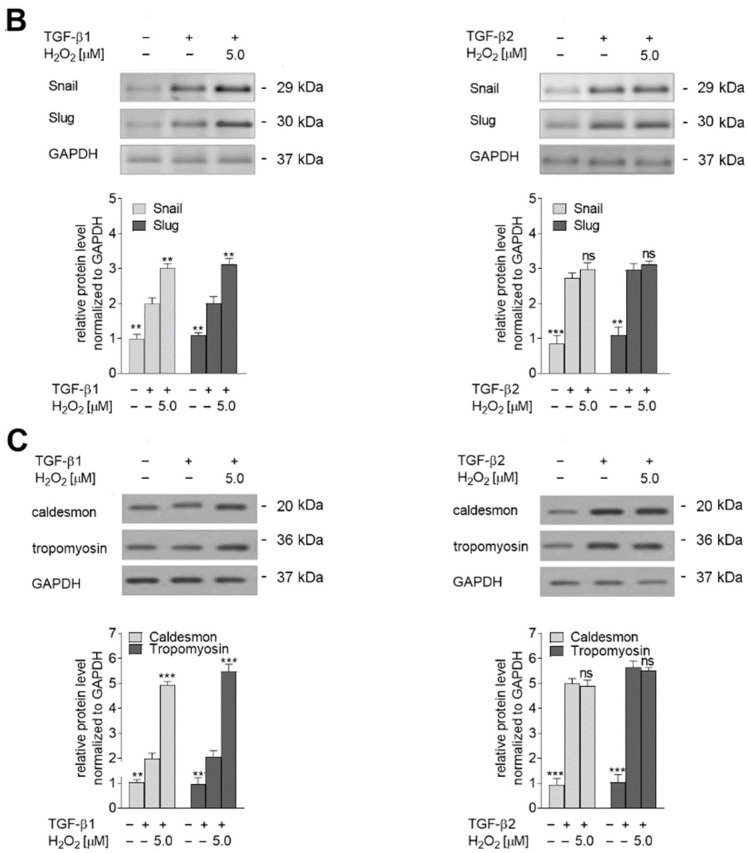
Co-stimulation by TGF-β1 or TGF-β2 and H_2_O_2_ induce later stages of EndMT in HMEC-1 cells. HMEC-1 cells were treated with TGF-β1 or TGF-β2 (10 ng/mL) and either 0 or 5.0 µM H_2_O_2_ for 48 h. (**A**) EndMT markers, (**B**) transcription factors, and (**C**) contraction markers levels were determined by Western blot analysis. The protein levels are normalized to those of GAPDH. The results are provided as means ± SD (*n* = 3); * *p* < 0.05, ** *p* < 0.01, *** *p* < 0.001, ns—not significant. The blots are representative of three independent experiments.

The behavioral alterations were accompanied by the modulation of endothelial and mesenchymal markers (Figure 2A). H_2_O_2_ co-stimulation yielded more evident changes in TGF-β1-stimulated cells: 5 µM H_2_O_2_ resulted in a near 0.2-fold decrease of claudin and ZO-1, 0.5-fold upregulation of α-SMA and FSP-1, and 1.0-fold higher N-cadherin protein levels. Finally, 5 µM H_2_O_2_ co-treatment resulted in a 0.3-fold increase of Snail and Slug protein levels compared to cells treated with TGF-β1 alone (Figure 2B) and 2.5-fold higher levels of contractile protein makers such as caldesmon and tropomyosin (Figure 2C). In contrast, this oxidative stress resulted in a statistically insignificant modulation of these proteins in the TGF-β2-induced cells (Figure 2A–C, right panels). Treatment with only H_2_O_2_ (5 µM) alone resulted in modulation of caldesmon and tropomyosin levels, but the changes were usually about 50% lower than observed after TGF-β1 stimulation alone (Figure S1).

### 2.2. Oxidative Stress Induces Later Stages of EndMT in Snail-Transfected HMEC-1 Cells

**Figure 3:**
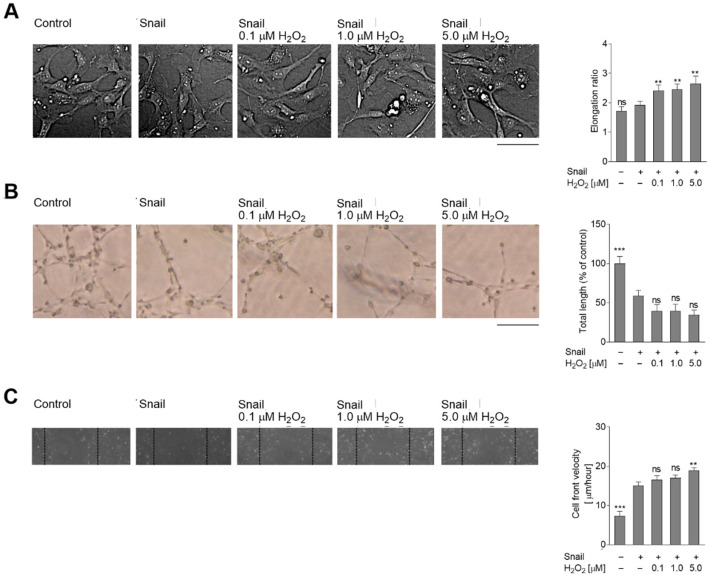
Snail-overexpressed cells were grown under oxidative stress, which induces the later stages of EndMT in HMEC-1 cells. HMEC-1 cells were treated with 0.1, 1.0, or 5.0 µM H_2_O_2_ for 48 h or only H_2_O_2_. (**A**) Morphology was analyzed by DIC microscopy. At least 50 cells were measured to calculate the elongation ratio. Representative images are shown, with the graph displaying means (*n* = 3) ± SD; ** *p* < 0.01, ns—not significant. Scale bar 100 µm. (**B**) Capillary formation was analyzed in Matrigel™. An appropriate number of cells were placed on Matrigel™, and after six hours, the tubes were observed under a microscope at ×40 magnification and photographed. Representative images are shown and quantitative analysis of mean tube lengths from three independent experiments was performed in ImageJ (*n* = 3) ± SD; *** *p* < 0.001, ns—not significant. Scale bar 100 µm. (**C**) The wound healing properties were analyzed. An appropriate number of cells was seeded on 12-well plates pre-coated with collagen I. After two days, a monolayer of confluent cell layer was wounded and images were captured 8 h later. Statistical significance in all experiments was marked: ** *p* < 0.01, *** *p* < 0.001, ns—not significant.

It is known that Snail overexpression induces the early stages of EndMT in HMEC-1 cells [15,16]. The present study examines whether oxidative stress enhances Snail-dependent cell elongation (Figure 3A). Our findings indicate that whereas Snail-transfected cells were approximately 12% more elongated than control cells, oxidative stress enhanced this effect by between 40% (0.1 µM H_2_O_2_) and 50% (5 µM H_2_O_2_). The next part of the study examined the impact of oxidative stress on the behavior of Snail-overexpressed cells. The total length of the capillaries was 41% lower in Snail-transfected cells, and oxidative stress induction increased this effect by 20% (5 µM H_2_O_2_) (Figure 3B). Simultaneous analysis of cell movement presented increased migratory ability in H_2_O_2_-treated cells, ranging from 10% in 0.1 µM to 21% in 5 µM H_2_O_2_, compared to Snail-overexpressed cells (Figure 3C).

**Figure 4:**
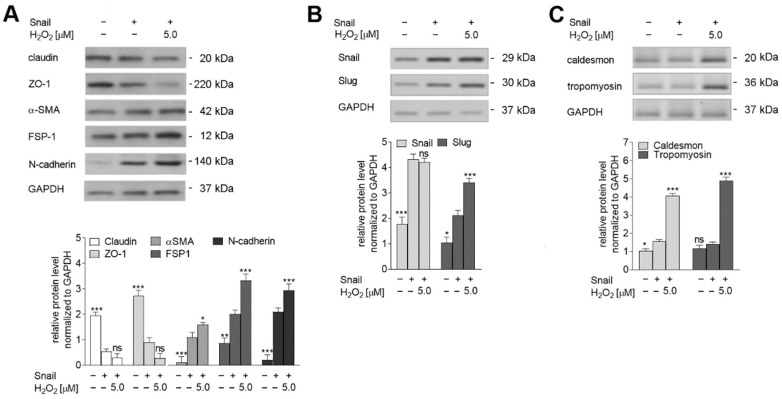
Snail-overexpressed cells were grown under oxidative stress, which induces the later stages of EndMT in HMEC-1 cells. HMEC-1 cells treated with TGF-βs (TGF-β1 or TGF-β2; 10 ng/mL) or overexpressed Snail were co-stimulated with 0 or 5.0 µM H_2_O_2_ for 72 h. (**A**) EndMT markers, (**B**) transcription factors, and (**C**) contraction markers levels were determined by Western blot analysis. The protein levels are normalized to those of GAPDH. The results are provided as means ± SD (*n* = 3); * *p* < 0.05, ** *p* < 0.01, *** *p* < 0.001, ns—not significant. The blots are representative of three independent experiments.

These changes in behavior were manifested by stronger modulation of endothelial and mesenchymal marker levels (Figure 4A). The effect of 5 µM H_2_O_2_ co-stimulation caused a near 0.5-fold decrease of claudin and a 0.7-fold reduction of ZO-1 levels, as well as a 0.3-fold increase in α-SMA, 0.9-fold increase in FSP-1, and more than 0.7-fold upregulation of N-cadherin levels. Among cells with Snail overexpression, Western blot analysis of EndMT transcription factors found that those treated with 5 µM H_2_O_2_ demonstrated no change in Snail protein level compared to untreated cells, but 0.8-fold upregulation Slug protein (Figure 4B). Regarding contraction makers, as in the TGF-β EndMT model, H_2_O_2_-treated cells demonstrated four-fold higher caldesmon levels and five-fold higher tropomyosin levels (Figure 4C).

### 2.3. Hydrogen Peroxide Regulates the Activation of MRTF-A in HMEC-1 Cells

As described above, the changes in cell morphology and behavior detected during mesenchymal transdifferentiation were associated with the modulation of contraction markers, which were enhanced by H_2_O_2_ co-stimulation. Their expression is known to be regulated by MRTF translocation to the nucleus and activation of the MRTF-SRF loop [5,17]. Therefore, we analyzed whether the stronger EndMT induction observed in oxidative stress is related to changes in MRTF activation.

**Figure 5:**
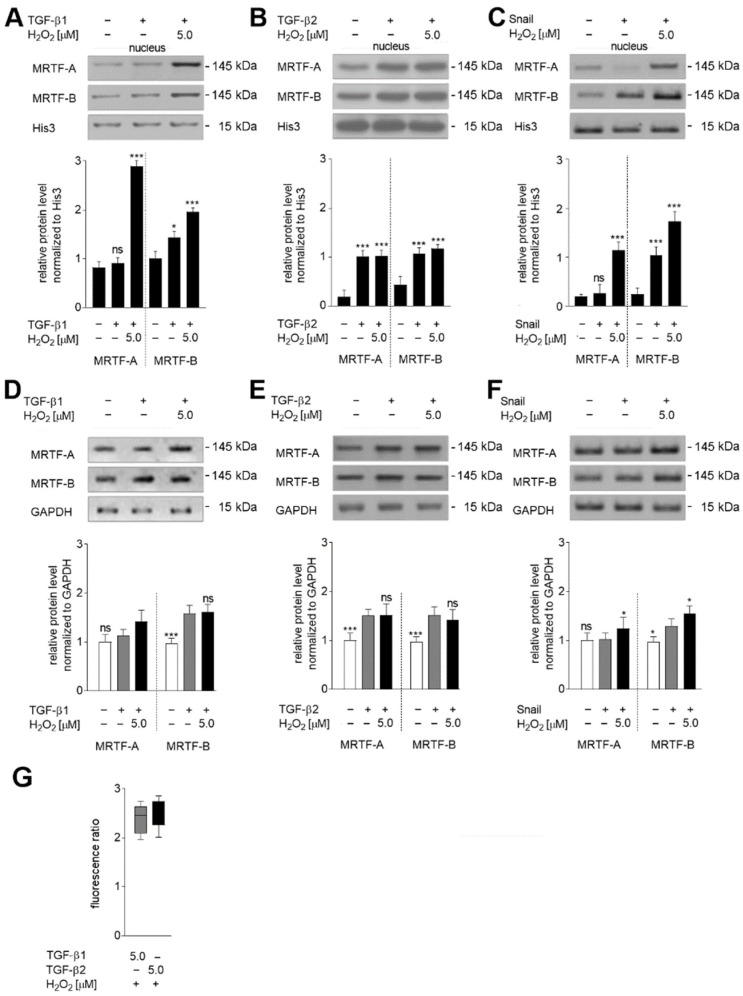
The oxidative stress increased the nuclear accumulation of MRTFs in EndMT-induced HMEC-1 cells. HMEC-1 cells treated with TGF-βs (TGF-β1 or TGF-β2; 10 ng/mL) or overexpressed Snail were co-stimulated with 0 or 5.0 µM H_2_O_2_ for 72 h. MRTF localization in nucleus fractions (**A**–**C**) and total MRTF protein levels (**D**–**F**) were determined by Western blotting. The protein levels are normalized to appropriate loading controls: nucleus: Histone 3 (His3); total: GADPH. Nuclear accumulation is shown as the percentage of total protein. The fluorescence ratio (**G**) was determined in by measurement of fluorescence of the H_2_O_2_ induced cells co-stimulated with TGF-β1 or TGF-β2 using the fluorescence of the cells stimulated only with TGF-β1 or TGF-β2 in a fluorescence microplate reader TECAN^®^ (excitation/emission 495/525 nm). The results are provided as means ± SD (*n* = 3); * *p* < 0.05, *** *p* < 0.001, ns—not significant. The blots are representative of three independent experiments.

As we showed previously, TGF-β1 treatment caused the translocation of about 50% of MRTF-B from the cytoplasm to the nucleus [5]. H_2_O_2_ co-stimulation resulted in higher activation of MRTFs, with about 2.9-fold higher levels of MRTF-B and 0.51-fold higher levels of MRTF-A being translocated to the nucleus (Figure 5A). In contrast, H_2_O_2_ co-stimulation of TGF-β2-treated cells, where both MRTFs were activated, i.e., 83% of MRTF-A and 85% of MRTF-B were detected in the nucleus did not induce statistically significant changes in whole protein level (Figure 5B). Oxidative stress resulted in a growth of MRTF-A protein in the nuclear fraction of Snail-overexpressed cells (Figure 5C); similar results were seen in the cells treated with TGF-β1 (Figure 5A).

Interestingly, analysis of total MRTFs expression revealed that cells co-stimulated by TGF-β1 and H_2_O_2_ demonstrated slight upregulation of MRTF-A (0.32-fold) (Figure 5D), with the levels reaching those observed in TGF-β2 treated cells (Figure 5E). The modulation level observed in Snail-overexpressed cells was similar to that in TGF-β1-stimulated cells (Figure 5F).

To confirm oxidative stress function’s effect, we measured the intracellular ROS generated in cells treated with 5 µM H2DCFH-DA. We detected a substantial (about 2.5× increase) fluorescence ratio calculated as the quotient of the fluorescence emitted by H_2_O_2_ and TGF-β1- or TGF-β2-stimulated cells to the emission of fluorescence in only TGF-β1- or TGF-β2-stimulated cells (Figure 5G).

### 2.4. Oxidative Stress Induces Late Stages of EndMT by Induction of TGF-β2 Upregulation

**Figure 6:**
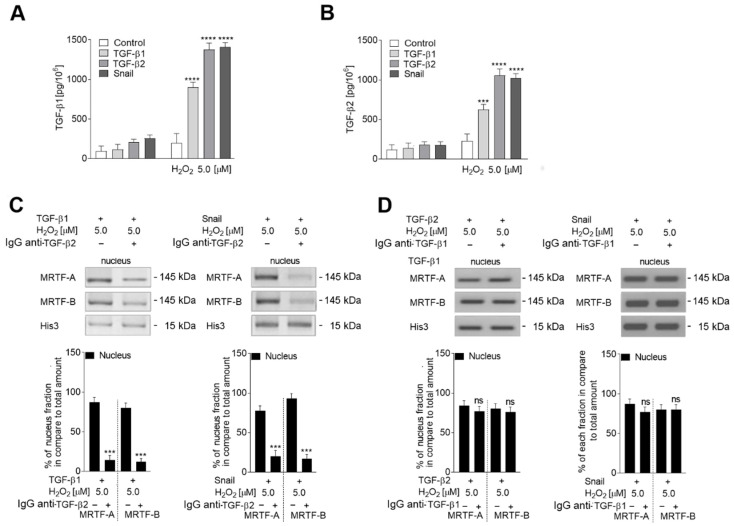
Oxidative stress induces TGF-β1 and TGF-β2 expression. HMEC-1 cells treated with TGF-βs (TGF-β1 or TGF-β2; 10 ng/mL) or overexpressed Snail were co-stimulated with 0 or 5.0 µM H_2_O_2_ for 72 h and the growth factor levels in conditioned medium were determined by Western blot (**A**,**B**). the TGF--β2 or TGF-b1 was depleted from conditioned medium as described in the text and MRTF protein levels in nucleus (**C**,**D**) were determined by Western blotting The protein levels are normalized to Histone 3 (His 3) and representative images are shown with the graph displaying means (*n* = 3) ± SD; **** *p* < 0.0001, *** *p* < 0.001, ns—not significant.

To explain the MRTF-A activation observed under oxidative stress, the TGF-β1 and TGF-β2 levels were studied in EndMT-induced cells exposed to H_2_O_2_. The levels of both TGF-β1 and TGF-β2 were measured in the conditioned medium from the EndMT-induced and H_2_O_2_-stimulated cells. It was found that TGF-β1 and TGF-β2 levels were higher when EndMT-stimulated cells were grown under oxidative stress conditions. In particular, secretion of TGF-β1 was found to be more than 7.3-fold higher in Snail-overexpressed cells, or those stimulated with TGF-β1 and treated with 5 µM H_2_O_2_, and about 6.8-fold higher than seen in cells treated with only TGF-β1 (Figure 6A). In contrast, the TGF-β2 level was approximately 6.75× higher in TGF-β2-stimulated or Snail-overexpressed cells maintained under oxidative stress and eight-fold higher than in cells treated with TGF-β2 alone (Figure 6B).

To confirm that those changes are not affected by the cross-reaction between antibodies that recognized TGF-β1 and TGF-β2. Anti-TGF-β2 antibodies did not bind to any of the bands recognized by commercially available TGF-β1 antibodies in any of the samples. Similarly, anti-TGF-β1 antibodies did not bind to the samples where TGF-β2 protein was separated. To confirm the role of both TGF-βs in EndMT stimulation, each growth factor was removed from the conditioned medium by the tested growth factors, which were specifically recognized by antibodies. We found that depletion of TGF-β2 resulted in the downregulation of MRTFs and the reduction of MRTFs located in the nucleus (Figure 6C). In contrast, the results in the cells depleted of TGF-β1 were similar to those stimulated with TGF-β2 (Figure 6D).

### 2.5. The TGF-β2-RhoA-MRTF-A/B Axis Is Critical for Later Stages of EndMT

**Figure 7:**
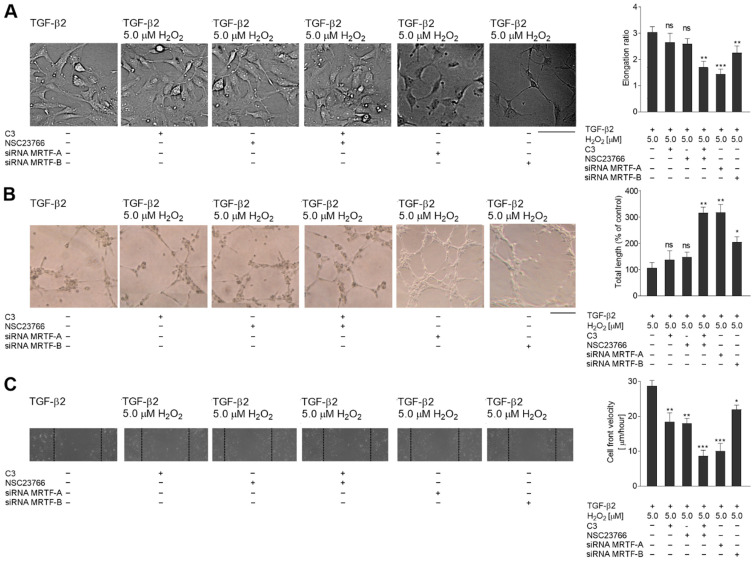

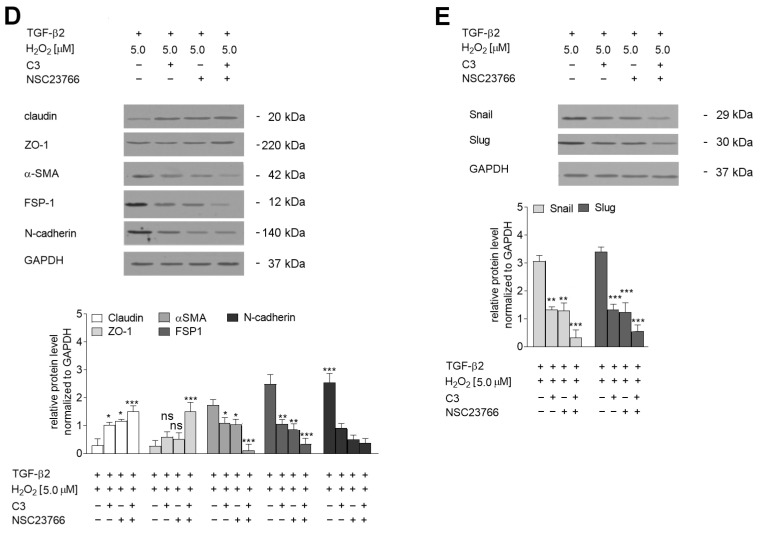
The TGF-β2-Rho-A/Rac1-MRTF-A axis plays a critical role in stimulating the later stages of EndMT. TGF-β-stimulated or Snail-overexpressed cells treated with H_2_O_2_ were incubated with RhoA or Rac1 inhibitors (C3, 2.0 μg/mL for 24 h and NCS23766, 100 nM for 6 h) before the end of TGF-β induction or siRNA MRTF-A(50 nM) or siRNA MRTF-B (50 nM). (**A**) Morphology was analyzed by DIC microscopy. At least 50 cells were measured to calculate the elongation ratio. Representative images are shown, with the graph displaying means (*n* = 3) ± SD; ** *p* < 0.01, ns—not significant. Scale bar 100 µm. (**B**) Capillary formation was analyzed in Matrigel™. An appropriate number of cells were placed on Matrigel™, and after six hours, the tube formation was observed under a microscope at ×40 magnification and photographed. Representative images are shown and quantitative analysis of mean tube lengths from three independent experiments were made in ImageJ (*n* = 3) ± SD; ** *p* < 0.01, ns—not significant. Scale bar 100 µm. (**C**) Next, the wound healing properties were analyzed. An appropriate cell number was seeded on 12-well plates pre-coated with collagen I. After two days, the monolayer of confluent cells was wounded and the images were captured 8 h later. Results are given as a standard error of mean cell front velocity (*n* = 3). (**D**) EndMT markers, (**E**) transcription factors levels. The protein levels are normalized to those of GAPDH. The results are provided as means ± SD (*n* = 3); * *p* < 0.05, ** *p* < 0.01, *** *p* < 0.001, ns—not significant. The blots are representative of three independent experiments.

Mesenchymal transdifferentiation stimulated by TGF-β2 is known to be dependent on the RhoA and Rac1 pathways [5]. Next, to check the influence of oxidative stress and TGF-β2 treatment on those pathways in HMEC-1 cells, they were treated with C3 transferase (2.0 μg/mL) and 100 nM NSC23766 inhibitors to block RhoA and Rac1, respectively. The inhibition of both pathways had cumulative effects. It resulted in strong inhibition of cell shape modulation (Figure 7A), angiogenesis ability (Figure 7B), and cell migration to collagen I (Figure 7C), which were dependent only on TGF-β2 stimulation.

These alterations of cell behavior were observed also on the molecular levels. Analysis of endothelial and mesenchymal marker levels (Figure 7D) usually correspond to the changes described above, i.e., inhibition of one molecular pathway limited the stimulation effect of TGF-β2 and H_2_O_2_ less than when both pathways were blocked (RhoA and Rac1). The level of Snail and Slug transcription proteins showed similar dependency (Figure 7E).

To confirm the role of MRTF-A and/or MRTF-B in the analyzed phenomenon, we decided to silence the MRTF-A or MRTF-B synthesis via interference RNA assay. Based on our previous studies [5], we applied 50 nM of siRNA that caused about an 85% decrease of MRTF-A and an 87% decrease of MRTF-B levels (Figure S2). The cell morphology observations showed that downregulation of MRTF-A in TGF-β2-stimulated cells inhibited cell elongation to the level observed in the control cells (Figure 7A vs. Figure 1A). In contrast, silencing of MRTF-B caused only slightly lower elongation cells than observed in the TGF-β2- and H_2_O_2_-stimulated cells. Capillary length and cell movement showed similar changes. Whereas MRTF-A silenced cells manifested behavior similar to control cells (Figure 7B,C vs. Figure 1B,C), a decrease of MRTF-B level resulted in only slightly modulation of the cells compared to TGF-β2- and H_2_O_2_-treated cells.

**Figure 8:**
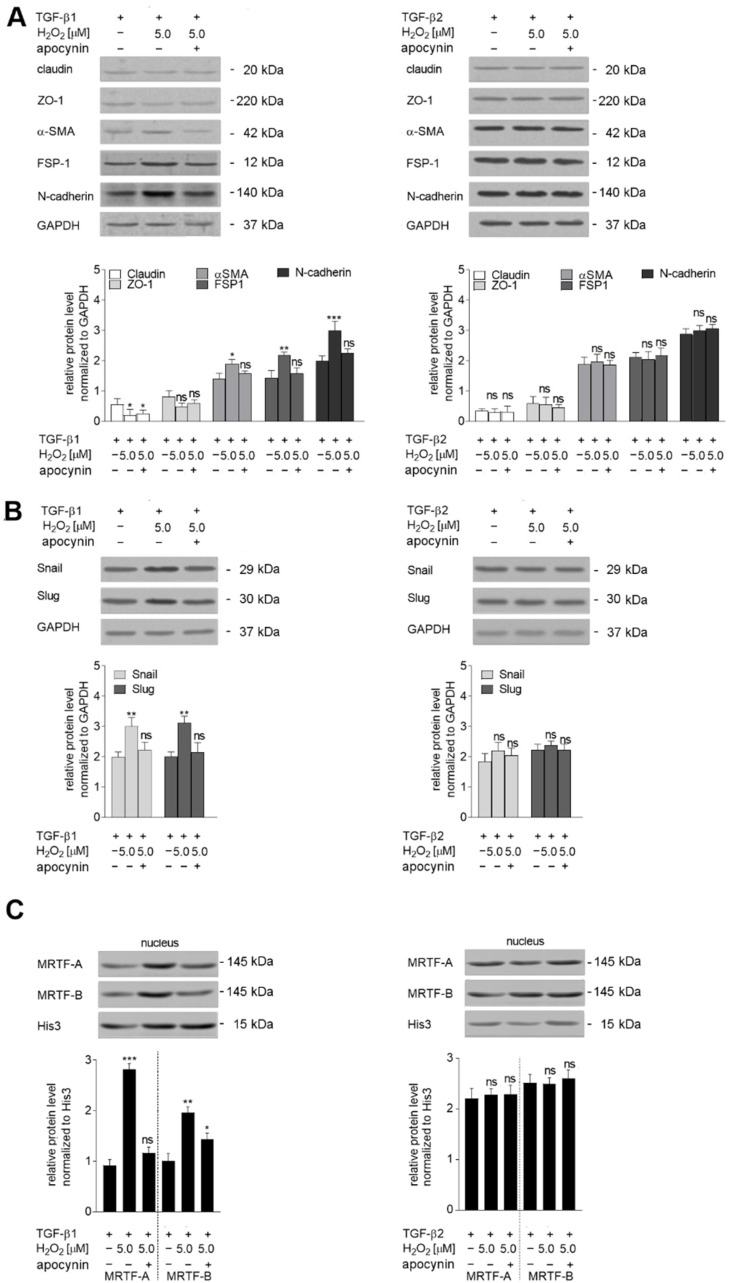
MRTF-A activation is crucial for stronger EndMT induction, HMEC-1 cells were treated with TGF-β1 or TGF-β2 (10 ng/mL), 5.0 µM H_2_O_2_ for 48 h, and 0.5 mM apocynin. (**A**) EndMT markers, (**B**) transcription factors Snail and Slug, and (**C**) nucleus MRTF-A and MRTF-B levels were determined by Western blot analysis. The protein levels are normalized to those of GAPDH. The results are provided as means ± SD (*n* = 3); * *p* < 0.05, ** *p* < 0.01, *** *p* < 0.001, ns—not significant. The blots are representative of three independent experiments.

Finally, to prove the role of oxidative stress deeply, we made a recovery model with the oxidative stress inhibitor apocynin. We have shown that in TGF-β1-stimulated cells, apocynin eliminated the H_2_O_2_ effect. In the lysate from those cells, endothelial and mesenchymal marker levels as well Snail and Slug levels were similar to what was detected in only TGF-β1-stimulated cells (Figure 8A,B). In contrast, in TGF-β2-stimulated cells, we did not detect any statistically significant alteration in the level of analyzed proteins (Figure 8C).

## 3. Discussion

It is well accepted that fibrosis is the final step of the pathological alterations related to many chronic inflammatory diseases. Although collagen deposition is a reversible part of wound healing under physiological conditions, the process may become uncontrolled under unfavorable circumstances. The main group of cells that secrete collagen in fibrotic tissues are myofibroblasts, transdifferentiated from a range of other cell types. Previously, it has been established that oxidative stress can regulate cell plasticity and generate myofibroblasts from endothelial cells during fibrosis progression [11,18]. Hence, the ability to control the endothelial transdifferentiation of ECs represents a very attractive goal in the search for new, more effective anti-fibrotic therapies.

Among the range of inflammation mediators, TGF-β family members are well-known inducers of EndMT in fibrotic tissues. Recently, Snail transcription factor [15,18] was described as an EndMT regulator, and it has been suggested that shear stress might regulate EndMT via Snail upregulation. Mesenchymal transdifferentiation may be a mechanism that initiates endothelial dysfunction during the inflammatory process. However, despite numerous studies, the precise mechanisms regulating transdifferentiation to early and/or late EndMT stages under oxidative stress conditions remain poorly understood.

The present study attempts to shed light on the role of TGF-βs in the changes that occur during EndMT in HMEC-1 cellular models exposed to oxidative stress. As HMEC-1 cells have been previously found to demonstrate different degrees of sensitivity to EndMT induction dependent on the presence of TGF-β1 and TGF-β2 [17], the present study analyzed both models and a previously described model, where the early stages of EndMT were induced by Snail overexpression. Co-stimulation by both TGF-βs and H_2_O_2_ decreased levels of endothelial protein markers (claudin, ZO-1). It increased the levels of mesenchymal markers (N-cadherin and vimentin) to those observed in cells treated with only TGF-β2. A similar effect was noted in Snail-overexpressed cells co-stimulated with H_2_O_2_. These changes in protein level correlated with dramatic differences in cell shape, capillary formation ability, and wound healing properties.

Additionally, strong upregulation of proteins characteristic for myofibroblast contraction was observed [5,6], and alterations in the levels of Snail, Slug, and ZEB1 transcription factors, which are critical for mesenchymal transdifferentiation [8,19]. Hence, it appears that oxidative stress can accelerate the EndMT stages in both tested TGF-βs, but the effect observed in the cells co-stimulated with TGF-β1 was more strongly marked. Comparable results were detected in Snail-overexpressed cells not subjected to oxidative stress, where only early stages of EndMT were observed [15,18].

The proteins belonging to the MRTF family regulate the expression of the protein associated with cytoskeleton reorganization and changes in cell morphology which occur during cellular transdifferentiation [20,21]. The activation of MRTFs via the Rho signaling pathway causes their accumulation in the nucleus, acting as SRF co-transcription factors [20]. The present study examining the localization and expression of MRTFs in EndMT-induced cells cultured in the presence of oxidative stress found that H_2_O_2_ co-stimulation affected the translocation of both MRTFs. However, in contrast to previous findings [5], the presence of H_2_O_2_ was not able to induce MRTF upregulation. Our study also revealed that TGF-β1 and H_2_O_2_ co-stimulation increased the nucleus pool of MRTF-A. This process is manifested by stronger alterations of cell shape and faster cells movement characteristics for later EndMT stages [17]. As we showed previously, EndMT in HMEC-1 cells was accompanied by high Snail and MRTFs upregulation and accumulation in the cell nucleus [5]. In contrast, TGF-β1 affected only the MRTF-B translocation and low Snail upregulation manifested by slightly marked behavioral alteration corresponding to early stages of EndMT [5]. Next, we supposed that both MRTF isoforms are critical for inducing the late EndMT stages via direct Snail synthesis regulation [17]. Here, we demonstrated that oxidative stress induction of MRTF-A correlated in TGF-β1-stimulated cells associated with later EndMT stages. The results confirmed our previous observation that both MRTFs are essential for strong mesenchymal transdifferentiations of HMEC-1 cells. However, further investigation is required to elucidate the degree of cooperation among MRTFs in the development of EndMT.

Our analysis showed that the activation of both the Rho-A and Rac1 pathways is critical for developing the later stages of EndMT. Only the inhibition of both of them might completely abrogate the effect of TGF-β or Snail-overexpression transdifferentiation. This is possible only when actin cytoskeleton polymerization may be inhibited. During that process, the free pool of G-actin is decreased in the cytoplasm by its polymerization to F-actin, which causes dissociation of the G-actin-MRTF complex. The release of MRTF seats to their translocation to the nucleus, where they act as SRF co-activators in abrogating the expression of numerous cytoskeleton and focal adhesion binding proteins [22,23].

The present study proposes mechanisms for the role of the TGF-β-RhoA/Rac1-MRTFA/B axis in the development of the later stages of EndMT, following activation by oxidative stress. Only TGF-β2 was able to induce MRTF-A and MRTF-B upregulation, activation, and nucleus accumulation, with the process being dependent on RhoA and Rac1 pathways. The activated MRTFs act as co-activators of numerous genes involved in the modulation of the actin cytoskeleton [24] and its ability to contract [23]. These modulations are characteristic of myofibroblast transdifferentiation and are associated with cell elongation and enhanced cell movement [25], thus hastening the later stages of EndMT and enhancing the development of fibrosis [26].

## 4. Materials and Methods

### 4.1. Reagents

All standard tissue culture reagents, including MCDB 131 medium, fetal bovine serum (FBS), and Penicillin-Streptomycin-Glutamine (100×), were obtained from Life Technologies (Paisley, UK). cOmplete™ EDTA-free Protease Inhibitor Cocktail was purchased from Roche (Basilea, Switzerland). TGF-β1 and TGF-β2 were obtained from R&D (Minneapolis, MN, USA). Enhanced Chemiluminescence (ECL) Western blotting substrate, M-PER Extraction Reagents, and NE-PER Nuclear and Cytoplasmic Extraction Kit were purchased from Thermo Scientific Pierce (Rockford, IL, USA). Small interfering RNA (siRNA) oligonucleotides were purchased from Dharmacon (Lafayette, CO, USA). Epithelial-Mesenchymal Transition (EMT were obtained from Cell Signaling Technology (Danvers, MA, USA). Goat anti-mouse antibodies, anti-rabbit antibodies conjugated with horseradish peroxidase, and mouse anti-GAPDH, -MRTF-A, and -MRTF-B were from Santa Cruz Biotech (Santa Cruz, CA, USA). X-fect was purchased from Clontech (Mountain View, CA, USA). Bradford, 30% acrylamide/bis 37.5:1 solution, ammonium persulfate (APS), 1,2-bis (dimethylamino) ethane (TEMED), glycine, and blotting membranes were obtained from Bio-Rad (Munich, Germany). Matrigel^TM^ was from Corning (Tewksbury, MA, USA). All other chemicals and solvents (including H_2_O_2_) were of the highest analytical grade and were purchased from Sigma-Aldrich (Steinheim, Germany).

### 4.2. Cell Lines

Human microvascular endothelial (HMEC-1) cells, a well-established microvascular endothelial cell line, were obtained as a gift from Kathryn Kellar, Center for Disease Control and Prevention, Atlanta, GA, and maintained in MCDB 131 medium supplemented with 10% (*v*/*v*) FBS, streptomycin (100 μg/mL), penicillin (100 units/mL), glutamine (2 mM), epidermal growth factor (EGF; 10 ng/mL), and hydrocortisone (1 μg/mL). All cell lines were cultured as a monolayer at 37 °C in a humidified atmosphere with 5% CO_2_ and routinely tested and confirmed as mycoplasma-free.

To simulate EndMT development, HMEC-1 cells were treated with 10 ng/mL TGF-β1 or TGF-β2 (10 ng/mL) for 48 h and/or 0.1, 1.0, or 5.0 µM H_2_O_2_ for two days. Optionally, cells were transfected with an expression vector encoding Snail (Snail-pcDNA3.1) (gifts from Muh-Hwa Yang, Ph.D., Institute of Clinical Medicine, National Yang-Ming University, Taipei, Taiwan) or an empty vector (pcDNA3.1) as a control transfection using the X-fect reagent according to the manufacturer’s protocol. In experiments with siRNA, oligonucleotides were added for 33 h. Inhibitors of small G proteins, RhoA (2.0 μg/mL C3 transferase) and Rac1 (100 nM NSC23766), were added for 24 and 6 h, respectively, before the end of TGF-β induction. In the recovery experiments apocynin (0.5 mM) was added to the growing cells.

### 4.3. siRNA Silencing

Four siRNAs targeting human MRTF-A or MRTF-B were applied, or a negative control siRNA, as described previously [5,17]. All siRNAs were transfected into cells using an X-fect reagent according to the manufacturer’s protocol. The siRNA treatment was performed for 33 h or 15 h after TGF-β1 or TGF-β2 stimulation.

### 4.4. Cell Morphology Analysis

The shape of the analyzed cells was examined by fluorescence microscopy (Olympus) as described previously [27]. Representative images were captured using an Olympus digital camera. Following this, changes in cell morphology were estimated by measuring the elongation ratio, i.e., the ratio of the longer to the shorter axis of the cells, using ImageJ software.

### 4.5. Enzyme-Linked Immunosorbent Assay

Conditioned medium (CM) was recovered from cells growing for 48 h and centrifuged to eliminate cells. TGF-β1 and TGF-β2 were quantified in the CM medium using sandwich enzyme-linked immunosorbent assay (ELISA) kits (Human TGF-β 1 and Human TGF--β 2 Quantikine ELISA Kits, R&D Systems, Minneapolis, MN, USA) according to the manufacturer’s instructions. The optical density of each reaction was measured at 450 nm using a microplate reader (Bio-Rad, Hercules, CA, USA) and corrected against absorption at 570 nm.

### 4.6. Western Blotting

The cell extracts were prepared as described elsewhere. Briefly, whole-cell extracts were prepared from exponentially growing cells by lysis in M-PER Mammalian Protein Extraction Reagent supplemented with Halt Protease Inhibitor Cocktail according to the manufacturer’s instructions. The extracts were collected, aliquoted, and stored at −80 °C until protein quantification with BCA Protein Assay Kit according to the manufacturer’s protocol. The nuclear and cytoplasmic protein fractions were analyzed using NE-PER Nuclear and Cytoplasmic Extraction Reagents according to the manufacturer’s protocols. Proteins from total lysates or nuclear or cytosolic fractions (30 µg) were separated by 10% sodium dodecyl sulfate-polyacrylamide gel electrophoresis (SDS-PAGE; Bio-Rad, Hercules, CA, USA) and electroblotted (120 min, 200 mA, 4 °C) onto nitrocellulose membranes (Bio-Rad). The membranes were blocked for two hours with Tris-buffered saline (TBS) containing 5% BSA at room temperature and incubated with an appropriate number of primary antibodies overnight at 4 °C. The membranes were then washed, incubated with anti-rabbit or anti-mouse horseradish peroxidase-conjugated secondary antibodies for one hour at room temperature, and further processed chemiluminescence detection using Pierce ECL Western Blotting Substrate and Kodak BioMax Light Film. The developed films were scanned using the HP Scanjet G4050 scanner, and the relative protein levels were quantified by the Gel Doc 2000 gel documentation system (Bio-Rad, Hercules, CA, USA). The background was subtracted, and the area for each protein peak was determined. Protein levels were normalized using an appropriate loading control.

### 4.7. Tube Formation Assay

In vitro, capillary-like tube formation was assessed using Matrigel™ according to the manufacturer’s instructions, as described previously [28]. The cells were first incubated at 37 °C for 60 min. Following this, the cells (5 × 10^4^/mL) in a complete cell culture medium were seeded onto 24-well Matrigel™-coated plates. After a 10-h incubation, the cells were observed under a phase-contrast microscope, and capillary-like structures were imaged using an Olympus digital camera. The formation of capillary tube-like networks was examined using ImageJ software. The total lengths of the capillaries were measured and are shown in the graphs.

### 4.8. Wound Healing

Confluent cells growing in 12-well plates were starved for four hours in an FBS-free medium [29]. The cell monolayer was wounded by scraping across the cells with a 200 L pipette tip; the cells were then rinsed twice with FBS-free medium and left in the medium supplemented with 1% BSA for 24 h. After wounding, the images were captured immediately (time = 0) and then eight hours later and stored for analysis. The migration of cells into the denuded area was visualized using an inverted Nikon phase-contrast microscope (400× magnification) and a digital camera (Olympus IX81, San Jose, CA, USA). Cell migration was quantified by image analysis of a minimum of four randomly selected fields of view of the denuded area. The mean wound area is expressed as a percentage of recovery (% R) from three identically treated plates using the equation: %R = [1 − (Tt/T0)] × 100, where T0 is the wounded area at 0 h and Tt is the wounded area eight hours post-injury.

### 4.9. Intracellular ROS Determination

Intracellular ROS generated in cells upon treatment by H_2_O_2_ were evaluated by measuring the oxidation of H2DCFDA-AM. Cells were seeded on 96-well microplates and treated as usually. Finally, the H2DCFH-DA was added to the culture medium (5 µM final concentration) and incubated for 30 min in dark at 37 °C. Then cells were washed twice with PBS, then 0.2 mL fresh PBS was added to each well, and the fluorescence was measured using a fluorescence microplate reader TECAN^®^ (excitation/emission 495/525 nm). The results were shown as a ratio of fluorescence to fluorescence emitted by the unstimulated control cells.

### 4.10. Statistical Analysis

Each experiment was repeated at least three times. Statistical significance was evaluated using Student’s *t*-test or one-way analysis of variance (ANOVA) followed by Tukey’s test with GraphPad Prism Software v 8.0. Differences between means were considered significant at *p* ≤ 0.05. The results are presented as means ± standard error, unless otherwise stated.

## 5. Conclusions

Our findings indicate that both the oxidative stress and the activation of MRTFs with synergistically regulated EndMT process seem to be new attractive targets for anti-fibrotic therapy that can inhibit the development of numerous fibrotic and cardiovascular diseases and even cancer.

## Data Availability

The data presented in this study are available upon request from the corresponding author (K.S).

## Supplementary Materials

The following supporting information can be downloaded at: https://www.mdpi.com/article/10.3390/ijms23042062/s1.
