# Supplementary material for: Oxidative Stress Enhances the TGF-β2-RhoA-MRTF-A/B Axis in Cells Entering Endothelial-Mesenchymal Transition
Source: Int J Mol Sci. 2022 Feb 13;23(4):2062. doi: 10.3390/ijms23042062 (PMC8879083; doi:10.3390/ijms23042062)
Supplement: Supplementary file 1 [file ijms-23-02062-s001.zip › ijms-1544465-supplementary.pdf]

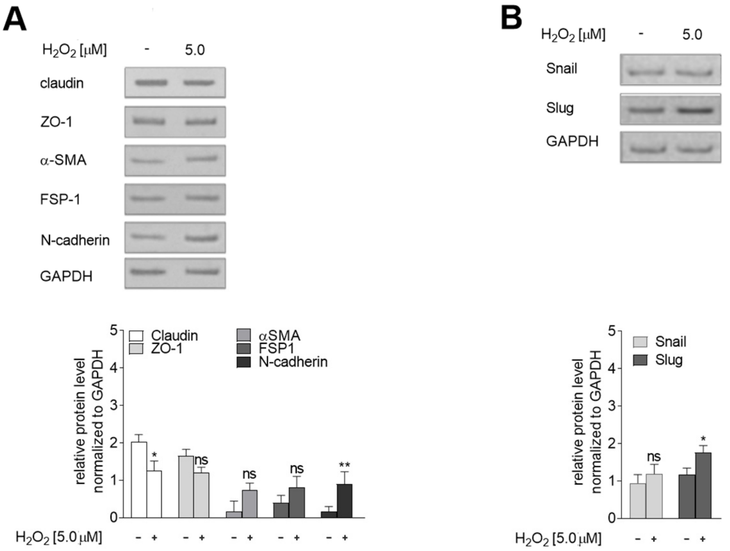

Figure S1. Stimulation by H_2O_2 induce later stages of EndMT in HMEC-1 cells. HMEC-1 cells were treated with 5.0 μM H_2O_2 for 48 hours. (A) EndMT markers, (B) transcription factors were determined by Western blot analysis. The protein levels are normalized to GAPDH. The results are provided as means ± SD (N=3); **p < 0.01, , ns – not significant. The blots are representative of three independent experiments.

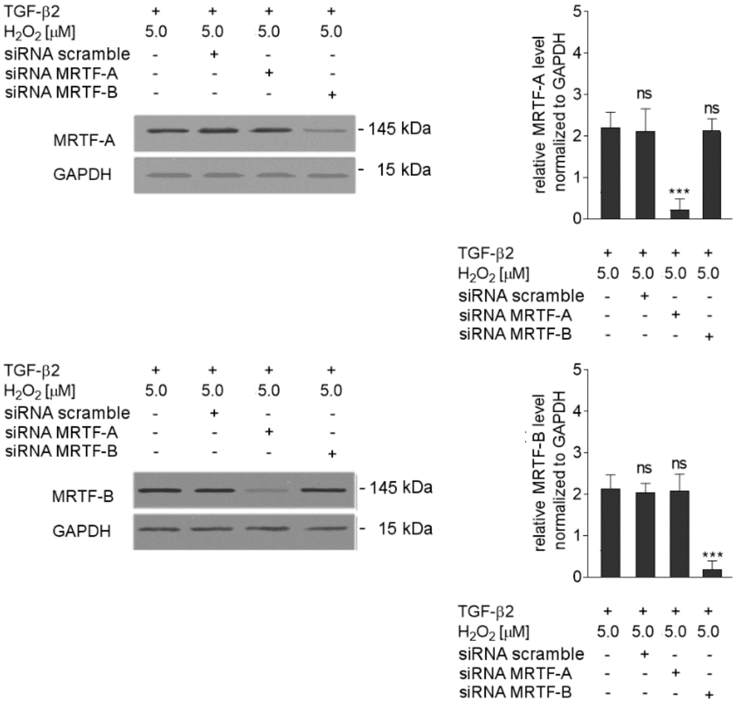

Figure S2 siRNA treatment caused decrease of MRTF-A or MRTF-B levels. HMEC-1 cells were treated with TGFb1 or TGFb2 and 5.0 μM H_2O_2 for 48 hours. The MRTF-A or MRTF-B were silenced with MRTF-A siRNA (50 nM) or MRTF-B siRNA (50 nM), respectively. MRTF-A or MRTF-B levels were determined by Western blot analysis. The protein levels are normalized to GAPDH. The results are provided as means ± SD (N=3); ***p < 0.001, ns – not significant. The blots are representative of three independent experimen
